# Epigenetic modifications of the *Zfp*/*ZNF423* gene control murine adipogenic commitment and are dysregulated in human hypertrophic obesity

**DOI:** 10.1007/s00125-017-4471-4

**Published:** 2017-10-24

**Authors:** Michele Longo, Gregory A. Raciti, Federica Zatterale, Luca Parrillo, Antonella Desiderio, Rosa Spinelli, Ann Hammarstedt, Shahram Hedjazifar, Jenny M. Hoffmann, Cecilia Nigro, Paola Mirra, Francesca Fiory, Pietro Formisano, Claudia Miele, Ulf Smith, Francesco Beguinot

**Affiliations:** 1 URT Genomics of Diabetes-IEOS, CNR & Department of Translational Medicine, Federico II University of Naples, Via Pansini 5, 80131 Naples, Italy; 2 Lundberg Laboratory for Diabetes Research, Department of Molecular and Clinical Medicine, Sahlgrenska Academy, University of Gothenburg, Gothenburg, Sweden

**Keywords:** Adipose tissue differentiation, Basic science, DNA methylation, Epigenetic regulation, Human, Insulin sensitivity and resistance, Pathogenic mechanisms, Transcription factors, Weight regulation and obesity

## Abstract

**Aims/hypothesis:**

Subcutaneous adipocyte hypertrophy is associated with insulin resistance and increased risk of type 2 diabetes, and predicts its future development independent of obesity. In humans, subcutaneous adipose tissue hypertrophy is a consequence of impaired adipocyte precursor cell recruitment into the adipogenic pathway rather than a lack of precursor cells. The zinc finger transcription factor known as zinc finger protein (ZFP) 423 has been identified as a major determinant of pre-adipocyte commitment and maintained white adipose cell function. Although its levels do not change during adipogenesis, ectopic expression of *Zfp423* in non-adipogenic murine cells is sufficient to activate expression of the gene encoding peroxisome proliferator-activated receptor γ (*Pparγ*; also known as *Pparg*) and increase the adipogenic potential of these cells. We investigated whether the *Zfp423* gene is under epigenetic regulation and whether this plays a role in the restricted adipogenesis associated with hypertrophic obesity.

**Methods:**

Murine 3T3-L1 and NIH-3T3 cells were used as fibroblasts committed and uncommitted to the adipocyte lineage, respectively. Human pre-adipocytes were isolated from the stromal vascular fraction of subcutaneous adipose tissue of 20 lean non-diabetic individuals with a wide adipose cell size range. mRNA levels were measured by quantitative real-time PCR, while methylation levels were analysed by bisulphite sequencing. Chromatin structure was analysed by micrococcal nuclease protection assay, and DNA-methyltransferases were chemically inhibited by 5-azacytidine. Adipocyte differentiation rate was evaluated by Oil Red O staining.

**Results:**

Comparison of uncommitted (NIH-3T3) and committed (3T3-L1) adipose precursor cells revealed that *Zfp423* expression increased (*p* < 0.01) in parallel with the ability of the cells to differentiate into mature adipocytes owing to both decreased promoter DNA methylation (*p* < 0.001) and nucleosome occupancy (nucleosome [NUC] 1 *p* < 0.01; NUC2 *p* < 0.001) in the 3T3-L1 compared with NIH-3T3 cells. Interestingly, non-adipogenic epigenetic profiles can be reverted in NIH-3T3 cells as 5-azacytidine treatment increased *Zfp423* mRNA levels (*p* < 0.01), reduced DNA methylation at a specific CpG site (*p* < 0.01), decreased nucleosome occupancy (NUC1, NUC2: *p* < 0.001) and induced adipocyte differentiation (*p* < 0.05). These epigenetic modifications can also be initiated in response to changes in the pre-adipose cell microenvironment, in which bone morphogenetic protein 4 (BMP4) plays a key role. We finally showed that, in human adipocyte precursor cells, impaired epigenetic regulation of zinc nuclear factor (*ZNF)423* (the human orthologue of murine *Zfp423*) was associated with inappropriate subcutaneous adipose cell hypertrophy. As in NIH-3T3 cells, the normal *ZNF423* epigenetic profile was rescued by 5-azacytidine exposure.

**Conclusions/interpretation:**

Our results show that epigenetic events regulate the ability of precursor cells to commit and differentiate into mature adipocytes by modulating *ZNF423*, and indicate that dysregulation of these mechanisms accompanies subcutaneous adipose tissue hypertrophy in humans.

**Electronic supplementary material:**

The online version of this article (10.1007/s00125-017-4471-4) contains peer-reviewed but unedited supplementary material, which is available to authorised users.

## Introduction

The worldwide increase in obesity is a major cause of the current epidemic of type 2 diabetes [1, 2]. However, obesity is not a homogeneous condition. Approximately 10–30% of obese individuals do not show metabolic complications. These individuals typically have an increased number of small adipocytes in their subcutaneous adipose tissue (SAT) and low visceral and other ectopic fat depots [3–5]. In addition, a similar proportion of non-obese individuals exhibit reduced insulin sensitivity and altered glucose metabolism [6]. At the molecular level, these human phenotypes remain incompletely characterised, generating uncertainties on how fat tissue expansion impacts the trajectory to type 2 diabetes.

Adipose tissue expansion is usually caused by an increase in adipocyte size (hypertrophy) and/or recruitment of new adipocytes from multipotent mesenchymal stem cells (MSCs) already in the stromal vascular compartment (hyperplasia) [7, 8]. Limited expandability and recruitment of new cells in SAT leads to prominent adipocyte hypertrophy, which is associated with ectopic accumulation of fat, functional dysregulation of SAT, low-grade chronic inflammation, decreased insulin sensitivity and enhanced oxidative stress [9–11]. In humans, SAT hypertrophy appears to be a consequence of impaired adipocyte precursor cell recruitment into the adipogenic pathway rather than a lack of precursor cells [12–15]. Although the underlying molecular mechanisms have only been partially elucidated, current evidence indicates that restricted adipogenesis in SAT predicts future development of type 2 diabetes independent of obesity [16]. The present understanding of SAT expansion in human obesity and diabetes is limited by incomplete understanding of the molecular basis of pre-adipocyte determination [17]. Recently, the zinc finger transcription factor known as zinc finger protein (ZFP) 423 was identified as a major determinant of pre-adipocyte commitment [17] and maintained white adipose cell function [18]. *Zfp423* expression is enriched in a number of adipogenic fibroblast cell lines compared with fibroblasts uncommitted to the adipocyte lineage. Although *Zfp423* levels are essentially unchanged during adipogenesis, ectopic expression of *Zfp423* in non-adipogenic murine cells is sufficient to activate expression of the gene encoding peroxisome proliferator-activated receptor γ (*Pparγ*; also known as *Pparg*) and increase the adipogenic potential of these cells [17, 19]. *Zfp423* knockout mice feature impaired development of both white and brown adipose tissue [17, 19].

The activity of ZFP42*3* in adipose precursor cells is repressed by the intracellular and secreted mediator WNT-inducible secreted protein 2 (WISP2). WISP2 production is significantly upregulated in the SAT of individuals with hypertrophic obesity, and is positively correlated to adipose cell size [20]. In the cytoplasm, WISP2 protein forms a complex with ZFP423 and prevents its translocation into the nucleus. Bone morphogenetic protein 4 (BMP4), a secreted protein and key regulator of the commitment of multipotent MSCs to the adipocyte lineage, dissociates this complex, allowing nuclear entry of ZFP423, thereby activating *Pparγ* transcription and commitment of precursor cells into the adipocyte lineage [12, 20].

Several studies have reported that epigenetic regulatory mechanisms are involved in the determination of multipotent precursor cells to form committed pre-adipocytes and the differentiation of pre-adipocytes to mature adipocytes [21]. Bioinformatic analysis of CpG islands in the promoter regions of obesity-related genes has identified regions with a high density of CpGs implicated in adipogenesis and inflammation, such as *Pparγ*, phosphatase and tensin homologue, leptin and tumour necrosis factor-α [22, 23]. Methylation of these CpG islands influences local chromatin structure and function, and participates in regulation of transcriptional activation of genes [24, 25].

Elucidation of the molecular mechanisms responsible for transcriptional regulation of *Zfp423* may improve the understanding of restricted adipogenesis in hypertrophic obesity. Here, we investigated whether *Zfp423* is epigenetically regulated and whether these events are involved in the restricted adipogenesis seen in humans with expanded subcutaneous adipose cells.

## Methods

Media, sera, insulin, TRIzol and SuperScript III were obtained from Invitrogen (San Diego, CA, USA), rosiglitazone from Alexis (Grünberg, Germany) and 5-azacytidine, 3-isobutyl-1-methylxanthine and dexamethasone from Sigma-Aldrich (St Louis, MO, USA). pCpGfree-Lucia, *Escherichia coli* GT115 cells, and Luciferase reporter assay kit were from InvivoGen (San Diego, CA, USA), SYBR Green from Bio-Rad (Hercules, CA, USA) and the DNA Methylation Kit from Zymo Research (Orange, CA, USA). Micrococcal nuclease (MNase), Dam^−^/Dcm^−^ *Escherichia coli* cells and HpyCH4IV, M.SssI, HhaI and HpaII enzymes were obtained from New England Biolabs (Ipswich, WI, USA). The DNA Purification Kit and pGEM-T Easy Vector were from Promega (Madison, WI, USA), the PCR Purification kit from Qiagen (Hilden, Germany), and the Big Dye Terminator v3.1Cycle Sequencing Kit from Applied Biosystems (Foster City, CA, USA).

### Cell culture and adipocyte differentiation

Mouse embryonic fibroblasts (3T3-L1, NIH-3T3) were obtained from the American Type Culture Collection (Manassas, VA, USA). These mycoplasma-free cell lines were grown in DMEM with 10% FCS. For adipocyte differentiation, see electronic supplementary material (ESM) Methods.

### Participants

This study is a secondary analysis of participants from the European network on Functional Genomics of Type 2 Diabetes (EUGENE2) consortium [26]. Adipose tissue-derived stromal vascular fraction (SVF) cells were obtained from 20 healthy, non-obese individuals whose recruitment and clinical phenotyping has previously been described [26]. The study was approved by the appropriate Institutional Review Boards. All participants gave informed consent.

Adipose tissue biopsies were obtained from abdominal SAT. Following careful dissection, adipose cells were digested with collagenase for 45 min at 37°C. After digestion, the suspension was centrifuged to obtain two phases: an upper (mature adipocytes) and a lower (SVF cells) phase. Adipocyte size was measured according to previously described procedures [13, 16]. SVF cells, in which we analysed *ZNF423* expression, were cultured in DMEM and Ham’s F-12 supplemented with 10% FBS and 0.002 mol/l glutamine as previously reported [13], in order to remove erythrocytes and inflammatory cells.

### RNA isolation and quantitative real-time PCR

RNA was isolated by TRIzol reagent according to the manufacturer’s protocol. RT-PCR of 1 μg of RNA was performed using SuperScript III. The cDNA obtained was used as a template for quantitative real-time PCR (qPCR), performed in triplicate using iQ SYBR Green Supermix on an iCycler real-time detection system (Bio-Rad). Relative quantification of gene expression was relative to the control (equal to 1) and was calculated according to the comparative ${2}^{-\Delta \Delta {\mathrm{C}}_{\mathrm{t}}}$ method based on the cycle threshold (C_t_) values of the target and housekeeping genes.

The primers used for *Ppia* (also known as *Cypa*), *Pparγ2*, *Fabp4* (also known as *Ap2*), *Glut4* (also known as *Slc2a4*), *Adipoq*, *Zfp423* and *ZNF423* gene expression in qPCR are reported in the ESM Table 1.

### MNase protection assay

Nuclei were isolated from both NIH-3T3 (5 × 10^5^ cells) and 3T3-L1 (5 × 10^5^ cells) and digested with 200 U of MNase for 20 min at 37°C. The purified DNA was subsequently amplified by qPCR. The percentage of nucleosome occupancy across the analysed regions was quantified by the ${2}^{-\Delta {\mathrm{C}}_{\mathrm{t}}}$ method using the undigested input as a normalising control, using NuPoP software (available from https://rdrr.io/bioc/NuPoP/; accessed November 2015).

### DNA methylation assessment

Genomic DNA was extracted using a DNA Purification Kit (Promega). Bisulphite treatment of extracted genomic DNA was performed using the EZ DNA Methylation Kit (Zymo Research). The bisulphite-converted genome was amplified by PCR using bisulphite-specific primers for *Zfp423* and *ZNF423* (see ESM Table 1 for the primers used). Bisulphite genomic sequencing was performed as previously reported [27]. DNA sequencing was performed on an ABI 3500 Automatic Sequencer using Big Dye Terminator v3.1 (Applied Biosystems). Bioinformatic analysis was carried out using EMBOSS CpGplot (available from www.ebi.ac.uk/Tools/seqstats/emboss_cpgplot/; accessed November 2015).

### In vitro methylation and luciferase reporter assay

The 5′-flanking region of *Zfp423* (−1324 to −764) was amplified by PCR and cloned into pCpGfree-Lucia (InvivoGen) luciferase reporter vector. Amplification of the reporter construct was performed using Dam^−^/Dcm^−^ *E. coli* cells. These cells were purchased from the New England Biolabs and are mycoplasma-free. The luciferase reporter vector was in vitro methylated by incubation with 1 unit/μg of M.SssI enzyme (which methylates all CpGs) or 1 unit/μg of HhaI (which methylates the cytosines of the sequence GCGC) and HpaII enzymes (which methylate the cytosines in the sequence CCGG) at 37°C for 1 h. Fully methylated, unmethylated and partially methylated *Zfp423* reporter vectors were transfected in NIH-3T3 cells. To normalise luciferase activity, a control plasmid encoding a Renilla luciferase gene was cotransfected into the cells. After 48 h, firefly and Renilla luciferase activity were assayed using a luciferase reporter assay kit (InvivoGen), according to the manufacturer’s instructions.

### Site-directed mutagenesis and luciferase reporter assay

*Zfp423* promoter (−1037/−1002) was amplified by PCR and cloned into the firefly luciferase reporter pCpGfree-promoter-Lucia vector (InvivoGen). A one-step PCR-based mutagenesis technique was used to generate site-specific mutation [28] and produce a mutated construct. One complementary pair of primers was designed that contained the desired mutation, replacing the cytosine at position −1016 with adenine. The wild-type and mutated constructs were transformed into *E. coli* GT115 cells. These cells were purchased from InvivoGen and are mycoplasma-free. In vitro methylation was performed using M.SssI methyltransferase following the manufacturer’s protocol (New England BioLabs). Unmethylated wild-type and mutated constructs were obtained in the absence of M.SssI. Methylation was confirmed by resistance to HpyCH4IV digestion (New England Biolabs). After 48 h, firefly and Renilla luciferase activity were assayed using a luciferase reporter assay kit, as reported in the previous paragraph.

### Statistical analysis

All experiments were performed three times for each determination, and results are shown as mean ± SD. Values for *p* between datasets were determined by two-tailed, unpaired Student’s *t* test. Significant *p* values are indicated as ***p < 0.001, **p < 0.01 and *p < 0.05. Correlation analysis was calculated using Pearson’s correlation coefficient.

## Results

### Promoter methylation reduces *Zfp423* expression in NIH-3T3 cells

**Fig. 1:**
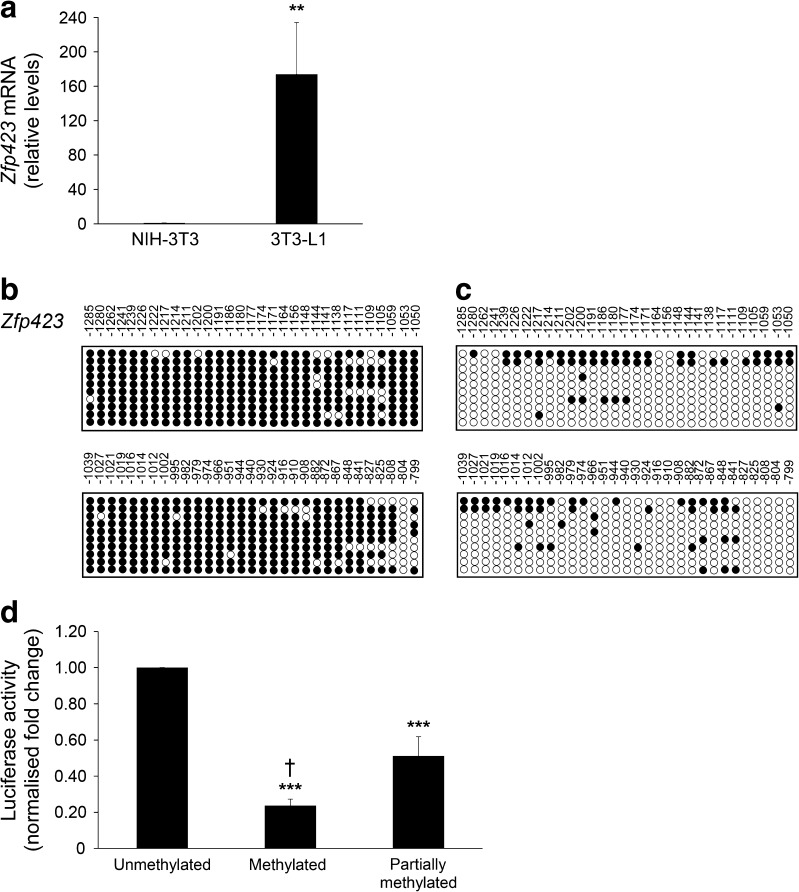
*Zfp423* mRNA expression and promoter methylation in NIH-3T3 and 3T3-L1 cells. (**a**) Total RNA was isolated from the cells, and *Zfp423* mRNA levels were assessed by qPCR. Data normalisation was achieved using the housekeeping *Ppia* gene as internal control. Results are the mean ± SD from three independent experiments. Statistical significance was tested by two-tailed Student’s *t* test (**p < 0.01 vs NIH-3T3). (**b, c**) Bisulphite sequencing analysis and assessment of *Zfp423* promoter methylation of individual methylated CpG sites were compared in NIH-3T3 (**b**) and 3T3-L1 (**c**) cells (methylation: 90.2 ± 2.1% NIH-3T3, 15.3 ± 2.7% 3T3-L1). Each PCR product was subcloned, and ten clones were analysed by bisulphite sequencing. Individual CpG sites at the *Zfp423* promoter, either methylated (black circles) or unmethylated (white circles), were aligned to their sequence position as indicated above the panels. Results are the mean ± SD from three independent experiments. Statistical significance was tested by two-tailed Student’s *t* test (*p* < 0.001 vs NIH-3T3). (**d**) Luciferase activity of the in vitro unmethylated, methylated or partially methylated *Zfp423* promoter reporter constructs in NIH-3T3 cells. Relative luciferase activity was normalised against the activity of a cotransfected internal vector. Results are the mean ± SD of three independent experiments. Statistical significance was tested by two-tailed Student’s *t* tests (***p < 0.001 vs unmethylated vector, ^†^ *p* < 0.05 vs partially methylated vector)

Using qPCR, we compared *Zfp423* mRNA expression in NIH-3T3 and in 3T3-L1 cells and found it to be barely detectable in the former and high in the latter (*p* < 0.01) (Fig. 1a). Importantly, there was no sequence variation of the *Zfp423* promoter in NIH-3T3 and 3T3-L1 cells (data not shown), suggesting that the differential expression observed had to be attributed to other mechanisms, including different epigenetic profiles. Furthermore, the expression of other key adipogenic marker genes was strongly silenced in NIH-3T3 cells (ESM Fig. 1).

To explore this, we subjected the *Zfp423* promoter region to bioinformatic analysis. EMBOSS CpGplot revealed a large 560 bp CpGi upstream *Zfp423* transcription start site, providing a potential basis for methylation control of *Zfp423* expression. We analysed the methylation status of the *Zfp423* CpGi by bisulphite sequencing in NIH-3T3 and 3T3-L1 cells, and found massive demethylation in the latter (methylation:15.3% vs 90.2% in NIH-3T3 cells; *p <* 0.001; Fig. 1b, c).

We then cloned the *Zfp423* promoter into the luciferase reporter vector (pCpGfree-basic-Lucia), which was either treated with M.SssI methylase and fully methylated, or partially methylated using HhaI and HpaII methylases. Digestion with the methylation-sensitive restriction enzyme HpyCH4IV enabled control of methylation level in the two conditions (data not shown). Luciferase activity in the constructs harbouring the fully and the partially methylated *Zfp423* promoters declined by 80% and 40%, respectively, compared with the unmethylated promoter (*p* < 0.001) (Fig. 1d). These results demonstrate that methylation regulates *Zfp423* promoter function in vitro.

### Nucleosome occupancy of *Zfp423* promoter is increased in NIH-3T3 compared with 3T3-L1 cells

**Fig. 2:**
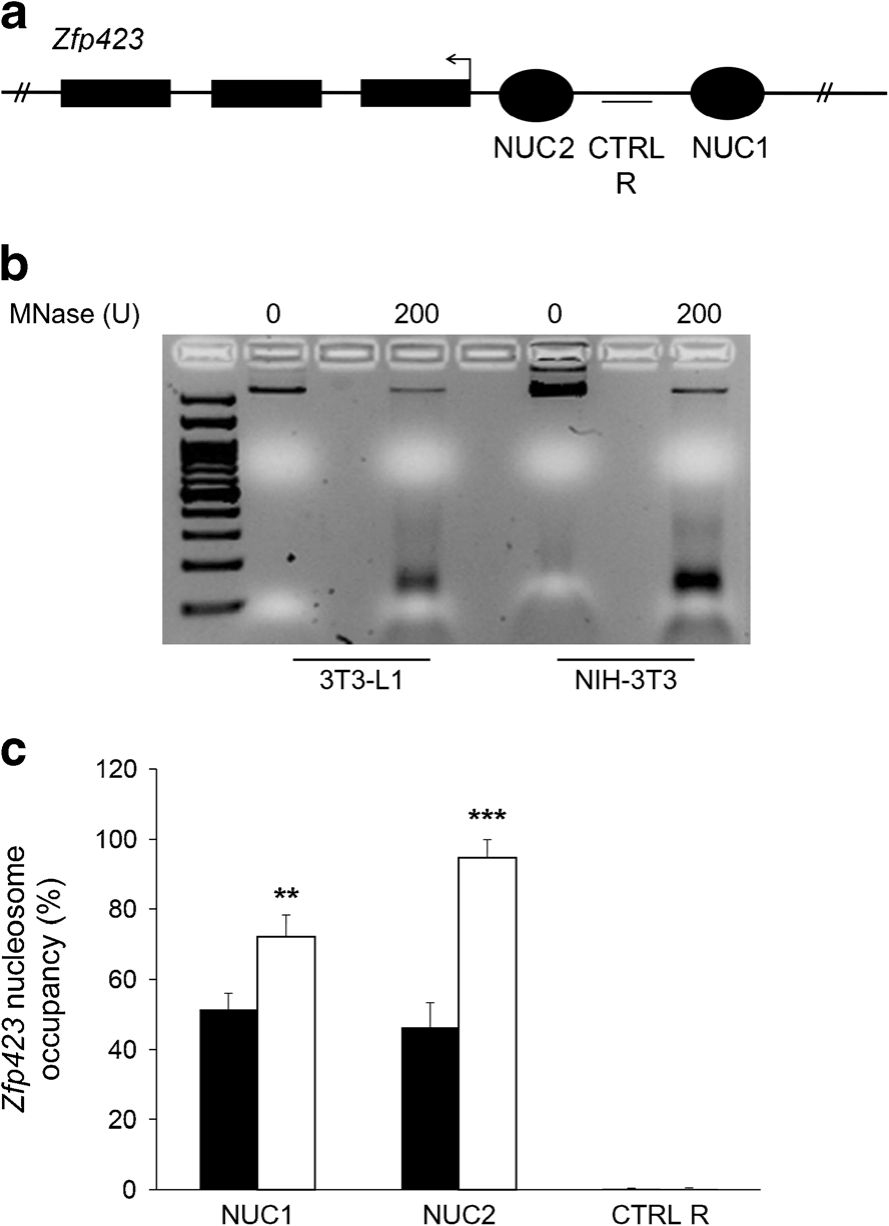
Nucleosome occupancy at the *Zfp423* promoter in 3T3-L1 and NIH-3T3 cells. (**a**) Schematic representation of the regions potentially occupied by nucleosomes at the *Zfp423* promoter in NIH-3T3 and 3T3-L1 cells. Genomic position: NUC1 chr8:87,960,746–87,960,945; NUC2 chr8:87,959,646-87,959,845; CTRL R chr8:87,960,121-87,960,322. (**b**) Genomic DNA was obtained by lysis of 3T3-L1 and NIH-3T3 cell nuclei and either digested with MNase or left untreated. (**c**) Percentage of nucleosome occupancy at two bioinformatically identified regions of the *Zfp423* promoter (NUC1, NUC2) and in the negative control region (CTRL R) was assessed by qPCR. Nucleosome occupancy across the analysed region was quantified by the ${2}^{-\Delta {\mathrm{C}}_{\mathrm{t}}}$ method using the undigested input as normalising control. Black bars, 3T3-L1 cells; white bars, NIH-3T3 cells. Results are the mean ± SD from three independent experiments. Statistical significance was tested by two-tailed Student’s *t* tests (**p < 0.01, ***p < 0.001 vs 3T3-L1 cells)

Based on NuPoP analysis, the *Zfp423* promoter exhibited several potential regions where nucleosome positioning featured a high prediction score (Fig. 2a), suggesting that differential *Zfp423* expression in NIH-3T3 and 3T3-L1 cells is also accompanied by a variation in nucleosome occupancy. To validate this and assess nucleosome occupancy at the best predicted regions, an MNase protection assay was performed and nucleosome positioning checked in mono-nucleosomal DNA by qPCR (Fig. 2b). The percentage of nucleosome occupancy at two such regions of the *Zfp423* promoter was significantly higher in NIH-3T3 cells (nucleosome [NUC]1% occupancy: 72.2 vs 51.5 in 3T3-L1 cells, *p* < 0.01; NUC2% occupancy: 94.6 vs 46.4 in 3T3-L1 cells, *p* < 0.001; Fig. 2c). No significant difference was observed in the CTRL R (negative control) region, where nucleosome positioning featured a low bioinformatic prediction score. Thus, in these cells, nucleosome occupancy of the promoter inversely correlates with *Zfp423* expression, suggesting that dynamic chromatin remodelling may also contribute to transcriptional regulation.

### 5-Azacytidine enhances *Zfp423* expression and allows differentiation of non-adipogenic NIH-3T3 cells

**Fig. 3:**
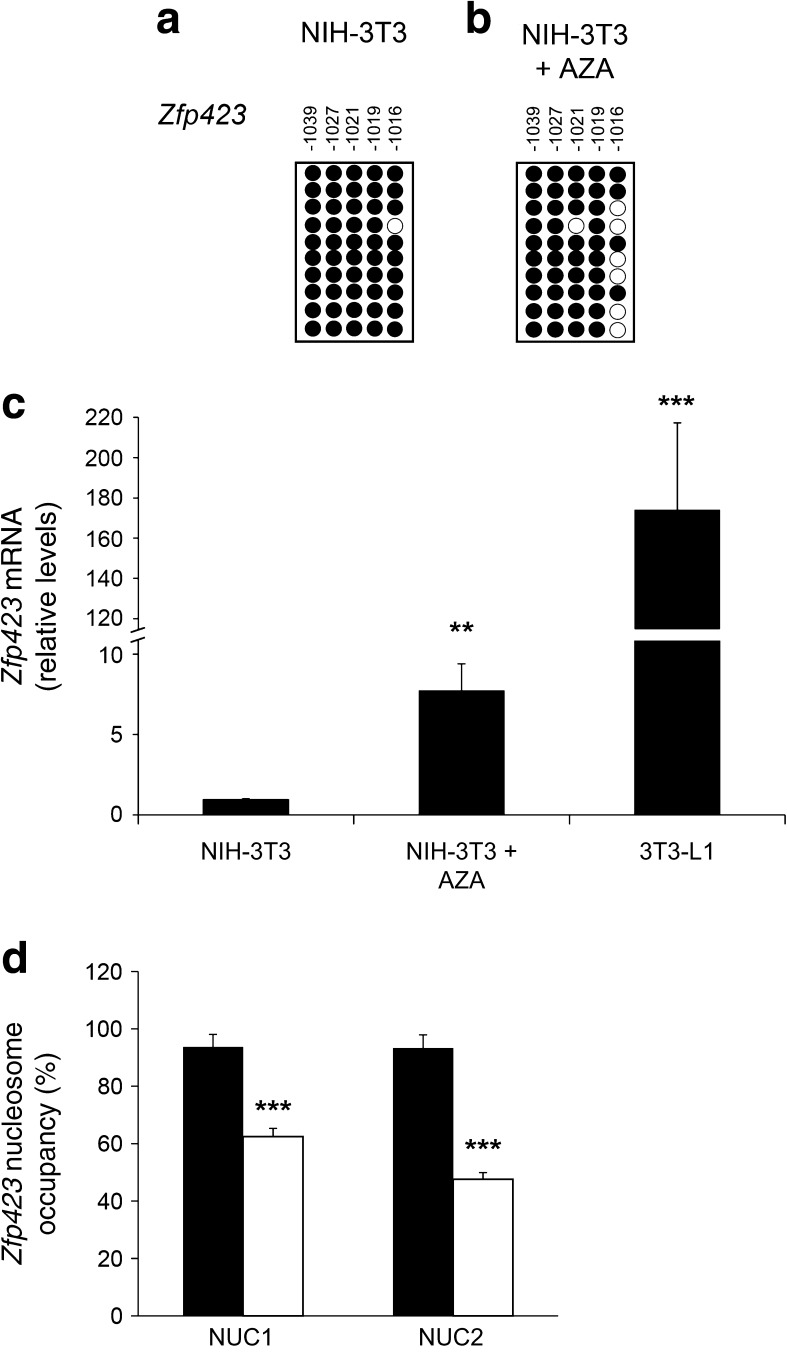
Effect of 5-azacytidine on *Zfp423* mRNA expression and nucleosome occupancy in NIH-3T3 cells. Cells were cultured in medium supplemented with 5-azacytidine (AZA). (**a, b**) Bisulphite sequencing analysis of DNA methylation and percentages of methylated −1016 CpG positions in the *Zfp423* promoter in NIH-3T3 and NIH-3T3 + AZA cells (methylation: −1016 CpG 90 ± 10% NIH-3T3, 40 ± 10% NIH-3T3 + AZA). Each PCR product was subcloned, and ten clones were analysed by bisulphite sequencing. The methylation profile of each CpG site in the *Zfp423* promoter, either methylated (black circles) or unmethylated (white circles), is aligned to its sequence position. Results are the mean ± SD from three independent experiments. Statistical significance was tested by two-tailed Student’s *t* tests (*p* < 0.01). (**c**) Expression of *Zfp423* mRNA was measured by qPCR in NIH-3T3, NIH-3T3 + AZA and 3T3-L1 cells. Data normalisation was achieved using the housekeeping *Ppia* gene as internal control. Results are the mean ± SD from three independent experiments. Statistical significance was analysed by two-tailed Student’s *t* tests (**p < 0.01, ***p < 0.001 vs NIH-3T3 cells). (**d**) Percentage of nucleosome occupancy was analysed by qPCR in the previously identified NUC1 and NUC2 regions of the *Zfp423* promoter. Assessment was performed in NIH-3T3 cells in the absence (black bars) or presence (white bars) of AZA. Results are the mean ± SD from three independent experiments. Statistical significance was tested by two-tailed Student’s *t* tests (***p < 0.001 vs NIH-3T3 without AZA)

To assess whether DNA methylation also regulates *Zfp423* expression in intact cells, we investigated the ability of the DNA methyltransferase inhibitor 5-azacytidine to remove the transcriptional block imposed on *Zfp423* in the NIH-3T3 cells. Incubating the cells with 5-azacytidine mainly affected methylation level at CpG position −1016 (40% methylation in exposed vs 90% in unexposed cells, *p <* 0.01; Fig. 3a, b); this was associated with a sixfold increase in *Zfp423* mRNA expression (*p* < 0.01; Fig. 3c). However, apart from the −1016 CpG, the overall methylation profile at the *Zfp423* promoter did not change in 5-azacytidine-treated cells (data not shown), providing a potential explanation for why mRNA expression levels are still lower than in 3T3-L1 cells (Fig. 3c). MNase protection studies revealed that 5-azacytidine significantly reduced nucleosome occupancy at the NUC1 and NUC2 regions (*p* < 0.001; Fig. 3d), further underlining the potential role of chromatin remodelling of the *Zfp423* regulatory region in transcriptional regulation.

**Fig. 4:**
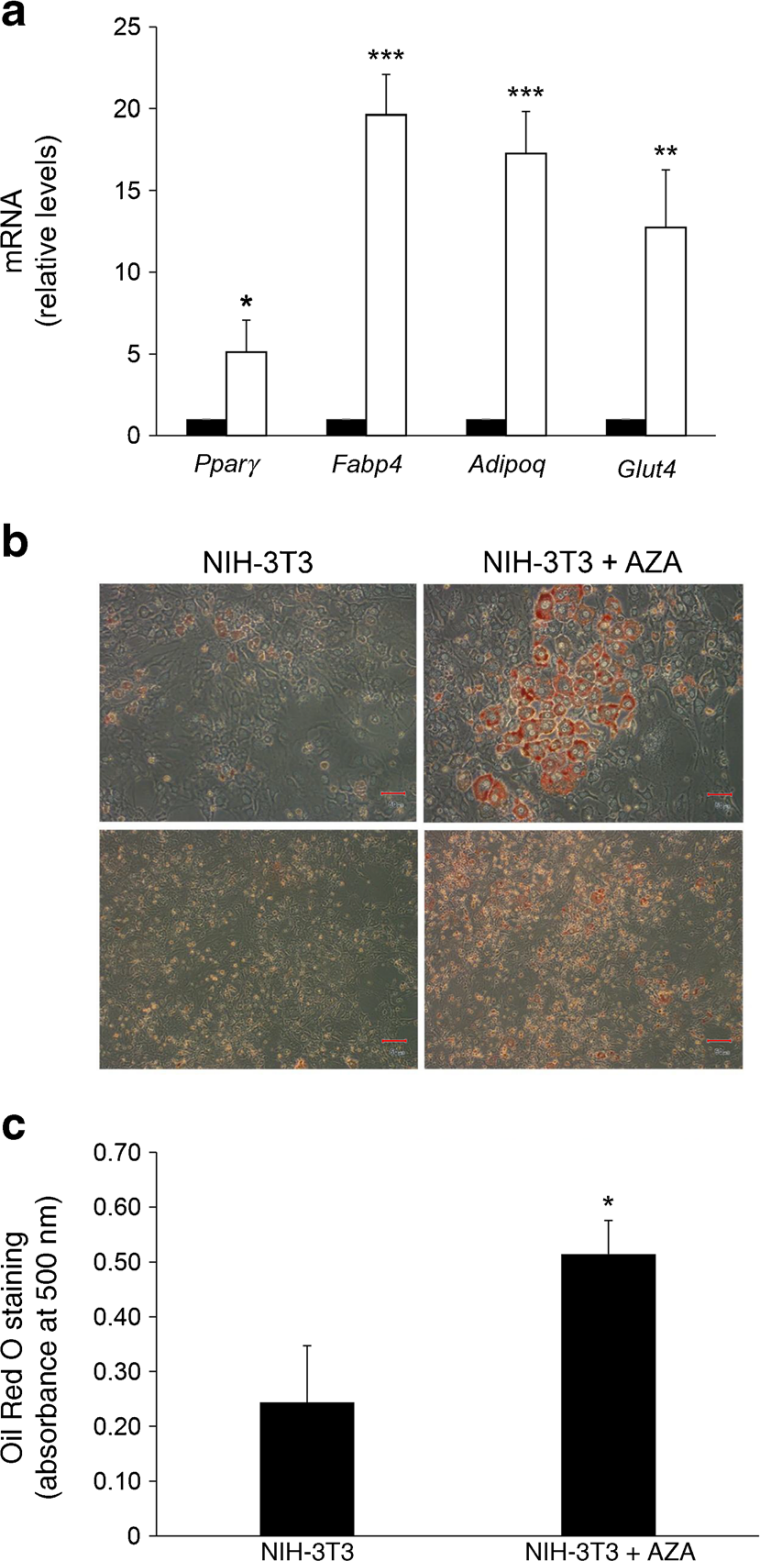
Effect of 5-azacytidine on NIH-3T3 cell adipogenic differentiation. NIH-3T3 cells were cultured in the absence (black bars) or presence (white bars) of 5-azacytidine (AZA). Gene expression and lipid accumulation were assessed 8 days after the induction of adipocyte differentiation. (**a**) The relative mRNA levels of *Pparγ*, *Fabp4*, *Adipoq* and *Glut4* were determined by qPCR. Black bars, NIH-3T3 cells; white bars, NIH-3T3 + AZA cells. Data normalisation was performed using the housekeeping *Ppia* gene as internal control. Results are the mean ± SD of three independent experiments. Statistical significance was established by two-tailed Student’s *t* tests (*p < 0.05, **p < 0.01, ***p < 0.001 vs NIH-3T3 without AZA). (**b**) At day 8, cells were fixed and stained with Oil Red O. Representative microphotographs are shown at two different magnifications (×20, ×10); scale bars, 50 μm. (**c**) Quantification of Oil Red O staining. Results are the mean ± SD of three independent experiments. Statistical significance was tested by two-tailed Student’s *t* tests (*p < 0.05 vs NIH-3T3 without AZA)

In parallel, 5-azacytidine robustly enhanced expression of *Pparγ* and the differentiation markers *Fabp4*, *Adipoq* and *Glut4* after induction of differentiation (Fig. 4a), accompanied by greater than twofold increased cytoplasmic accumulation of Oil Red O (Fig. 4b, c). Thus, in parallel with transcriptional activation, *Zfp423* promoter demethylation by 5-azacytidine also promoted differentiation of the non-adipogenic NIH-3T3 cell line.

### BMP4 promotes *Zfp423* expression by inducing promoter demethylation

**Fig. 5:**
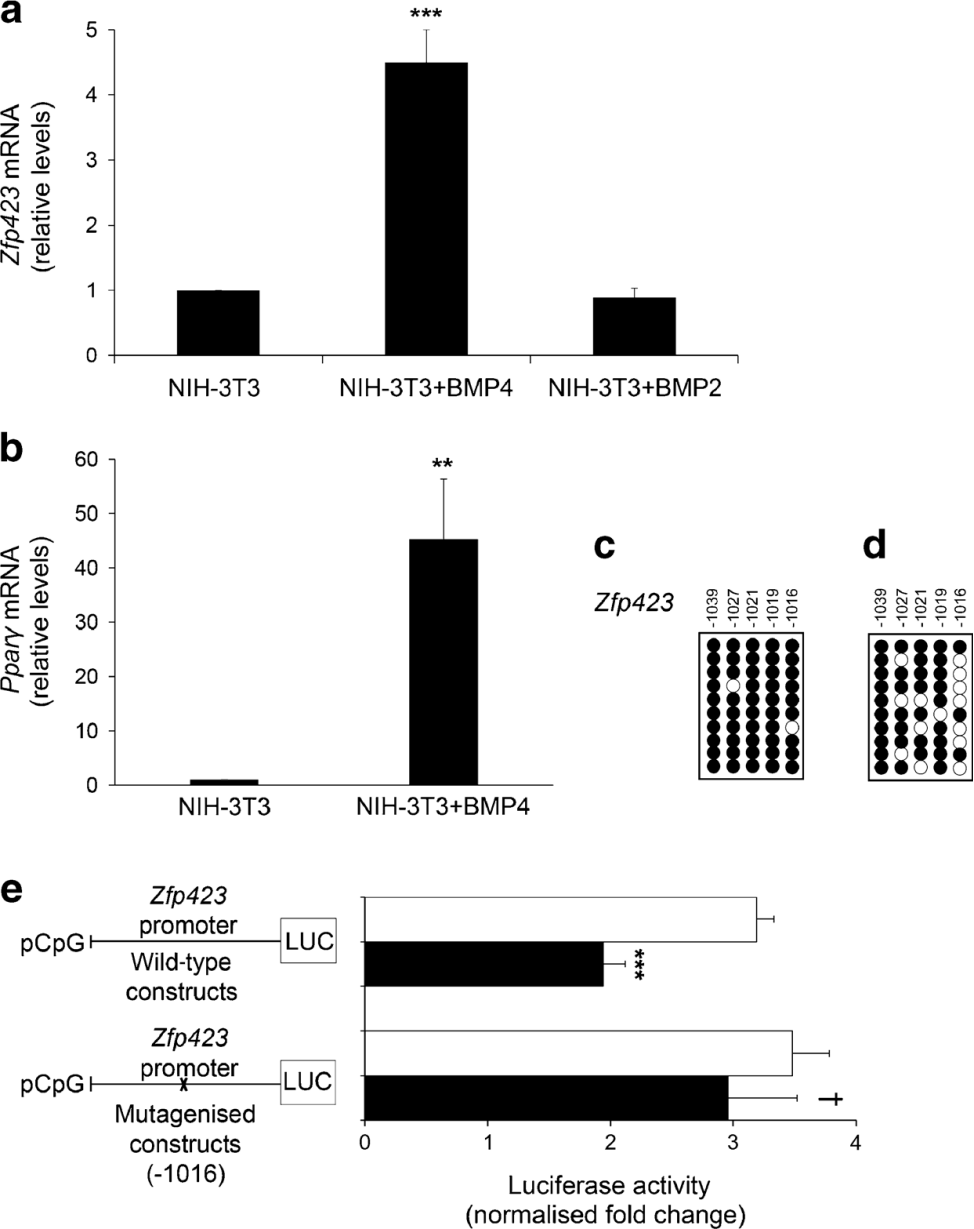
Effect of BMP4 on *Zfp423* mRNA expression and promoter methylation in NIH-3T3 cells. Cells were cultured in medium supplemented with 50 ng/ml BMP4 and 100 ng/ml BMP2. (**a, b**) Expression of *Zfp423* and *Pparγ* mRNA was measured by qPCR. Results are the mean ± SD from three independent experiments. Statistical significance was analysed by two-tailed Student’s *t* tests (**p < 0.01, ***p < 0.001 vs NIH-3T3). (**c, d**) Bisulphite sequencing analysis of the DNA methylation status and percentage of the methylated −1016 CpG position of the *Zfp423* promoter in NIH-3T3 cells on exposure to BMP4 (methylation −1016 CpG: 90 ± 10% NIH-3T3 [**c**], 30 ± 10% NIH-3T3 + BMP4 [**d**]). Each PCR product was subcloned, and ten clones were analysed by bisulphite sequencing. The methylation profile of each CpG site of the *Zfp423* promoter, either methylated (black circles) or unmethylated (white circles), was aligned to their sequence position. Results are the mean ± SD from three independent experiments. Statistical significance was tested by two-tailed Student’s *t* tests (*p* < 0.01 vs NIH-3T3 without BMP4). (**e**) Effect of mutagenesis at the −1016 CpG position of the *Zfp423* promoter. Disruption of CpG was performed by site-directed mutagenesis as described in Methods. Wild-type and mutated plasmids were treated with the DNA-methylase M.SssI and transfected into NIH-3T3 cells (black bars). White bars, untreated plasmids. Luciferase activity was normalised to *Renilla* luciferase activity. Error bars represent SD from three replicates. Statistical significance was tested by two-tailed Student’s *t* tests (***p < 0.001 vs wild-type unmethylated; ^†^ *p* < 0.05 vs wild-type methylated). LUC, luciferase reporter

BMPs are members of the transforming growth factor superfamily that play a key role in inducing adipocyte precursor cell commitment towards the adipogenic lineage; however, the molecular details of their action have been only partially elucidated. Adding BMP4 to the culture medium of NIH-3T3 cells enhanced the expression of both *Zfp423*, by almost fivefold (*p* < 0.001; Fig. 5a), and its downstream target gene *Pparγ* (*p* < 0.01; Fig. 5b), while *Zfp423* expression was only slightly reduced, albeit not reaching significance, by BMP2 treatment (*p =* 0.118; Fig. 5a). Bisulphite sequencing revealed that this BMP4-dependent change was accompanied by an almost threefold reduction in methylation at position −1016 in the *Zfp423* promoter (*p <* 0.01), reminiscent of that seen with 5-azacytidine treatment (Fig. 5c, d).

We therefore aimed to establish the functional significance of the −1016 dinucleotide for *Zfp423* promoter function by site-directed mutagenesis. After replacing the cytosine at position −1016 with adenine, the 35 bp fragment (−1037 to −1002) of the *Zfp423* promoter was cloned in a luciferase reporter vector (pCpGfree-promoter-Lucia). Luciferase activity was assayed in NIH-3T3 cells transfected with either the in vitro methylated or the unmethylated promoter. As shown in Fig. 5e, the −1016 mutation did not affect the activity of the unmethylated *Zfp423* promoter. However, it abolished the effect of methylation on promoter silencing, indicating that the −1016 dinucleotide modulates *Zfp423* transcription in vitro.

#### Pre-adipocyte *ZNF423* expression correlates with mature subcutaneous adipose cell size in humans

**Fig. 6:**
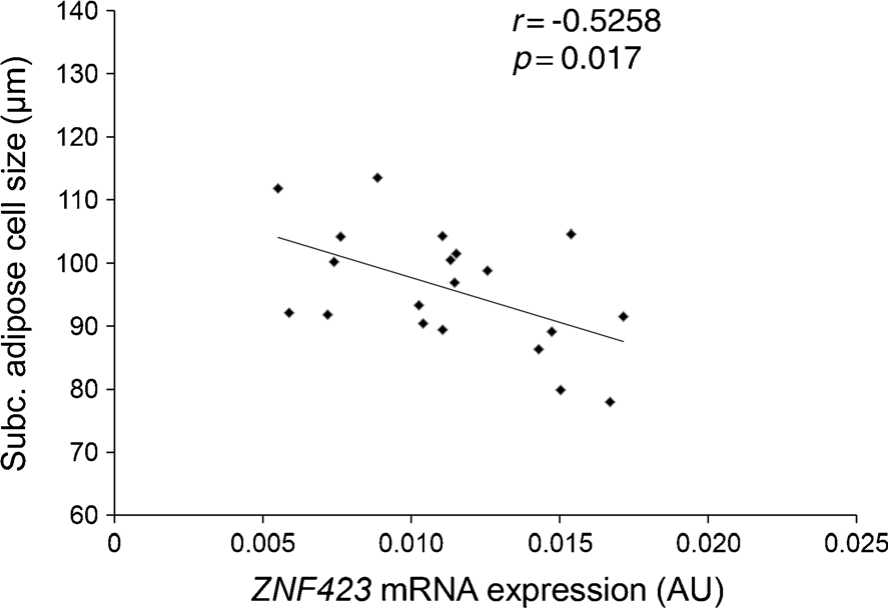
*ZNF423* expression in pre-adipocytes from non-obese individuals with different subcutaneous adipocyte size. *ZNF423* mRNA expression was quantified in pre-adipocytes obtained from the SVF of SAT from 20 healthy, non-obese individuals as described in Methods. Correlation was assessed by linear regression analysis. AU, absolute units; Subc., subcutaneous

To explore the significance of *Zfp423* expression in the development and function of human adipose tissue, we analysed transcription of human *ZNF423* (the human orthologue of *Zfp423*) [29] in pre-adipocytes from the SVF of 20 healthy, non-obese individuals. Participants were recruited as previously described [26] (see Table 1 for their clinical characteristics). The size of the subcutaneous adipose cells varied over a broad range even in these non-obese individuals, consistent with different adipogenic potential of the precursor cells. *ZNF423* mRNA was detectable in cells from all donors, and, importantly, expression in pre-adipocytes exhibited a significant negative correlation with the size of mature subcutaneous adipose cells from the same individuals (Fig. 6; *r* = −0.5258, *p* < 0.05). Thus, low *ZNF423* expression in adipose precursor cells is a marker of the donor’s subcutaneous adipocyte cell size, and thus adipogenesis and the development of inappropriate adipose cell hypertrophy, and associated with an insulin-resistant phenotype.

**Table 1:** Clinical characteristics of the study group

Variable	Mean ± SD
*N*	20
Age (years)	40.8 ± 7.9
BMI (kg/m^2^)	25.4 ± 2.6
Fat (%)	26.0 ± 6.7
Fat-free mass (kg)	57.4 ± 10.4
Cell size (μm)	95.9 ± 9.5
GIR/bw (mg/min)	9.1 ± 3.1
f-insulin (pmol/l)	348.0 ± 179.4
fb-glucose (mmol/l)	4.6 ± 0.5
OGTT p-glucose 2 h (mmol/l)	6.1 ± 1.8

fb-glucose, fasting blood glucose; f-insulin, fasting insulin; GIR/bw, glucose infusion rate/body weight; OGTT p-glucose, plasma glucose during OGTT

The central enhancer CpGi at the human *ZNF423* locus features >80% homology in mammals [29] and, based on site-specific mutagenesis studies in leukaemia cells, has been shown to be relevant for the functional regulation of both α and β *ZNF423* promoters [29]. In human pre-adipocytes, we observed that *ZNF423α* was the predominant isoform, while *ZNF423β* was barely detectable (ESM Fig. 2).

**Fig. 7:**
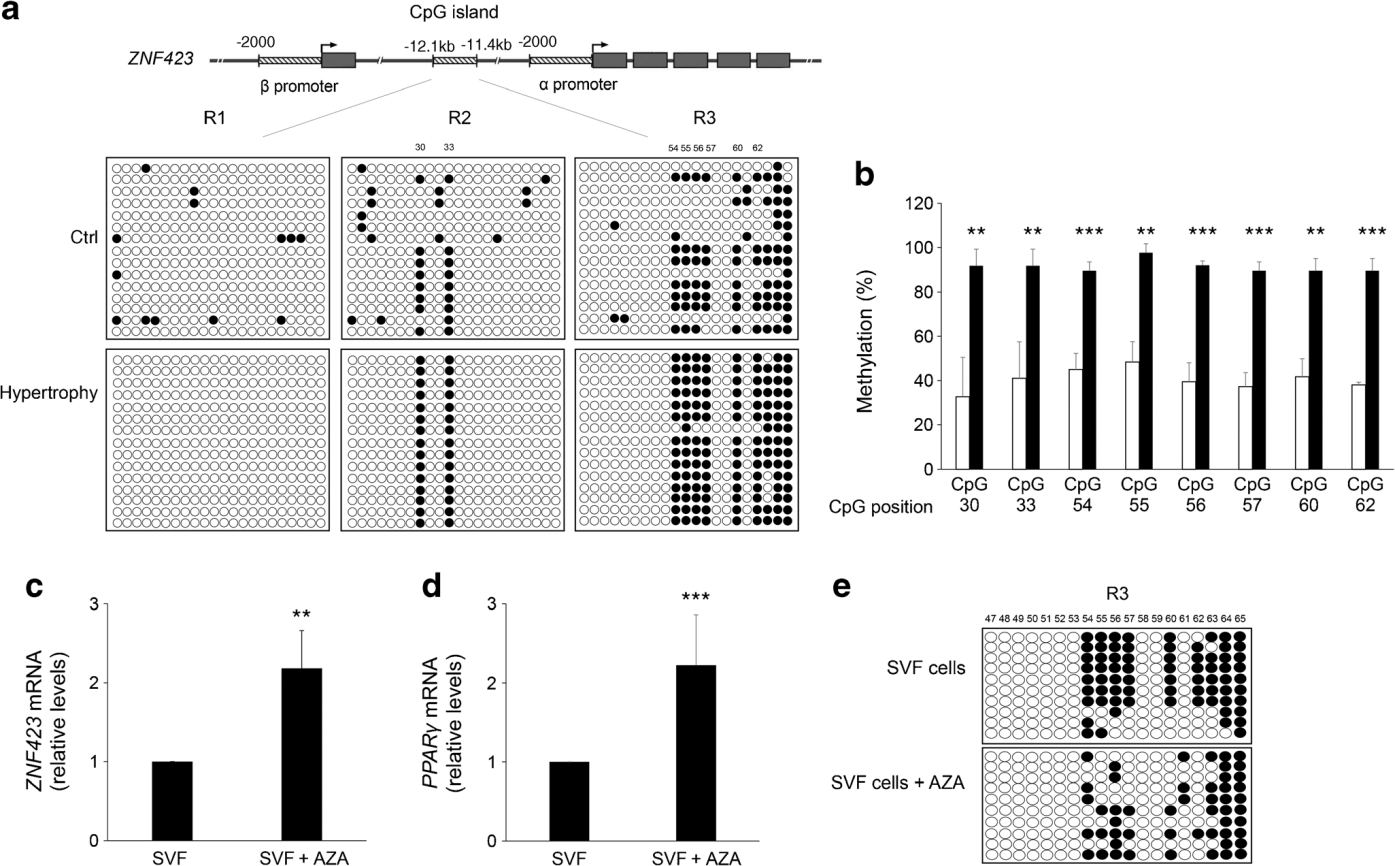
DNA methylation at the *ZNF423* promoter in SVF cells from non-obese individuals with subcutaneous adipocyte hypertrophy. Genomic DNA from pre-adipose SVF cells was obtained from three individuals analysed in Fig. 6 who exhibited the most extreme subcutaneous adipocyte hypertrophy (Hypertrophy) and three individuals with the smaller adipocyte size (Ctrl). Individual DNA preparations were exposed to bisulphite as described in Methods and individually analysed by PCR amplification of the CpGi at the *ZNF423* enhancer region (−12.1 kb; −11.4 kb from *ZNF423* gene transcription start site). PCR products were then individually subcloned and sequenced. (**a**) Methylation of 65 CpGs at the three *ZNF423* promoter regions (R1–R3) for 15 replica clones. White circles, unmethylated CpGs; black circles, methylated CpGs. A representative experiment with one individual with SAT hypertrophy and one control individual is shown (*n* = 3). (**b**) Quantification of the methylation levels at eight specific CpGs in individuals with the smallest size of subcutaneous adipocytes (white bars) or the largest adipocyte size (black bars). Error bars represent SD from three individuals per group. Statistical significance was tested by two-tailed Student’s *t* tests (**p < 0.01, ***p < 0.001 vs individuals with smallest adipocytes). (**c, d**) Expression of *ZNF423* and *PPARγ* mRNA was measured by qPCR. Results are the mean ± SD from three independent experiments. Statistical significance was analysed by two-tailed Student’s *t* tests (**p < 0.01, ***p < 0.001 vs SVF cells). (**e**) Methylation at the R3 region on *ZNF423* promoter for ten replica clones. White circles, unmethylated CpGs; black circles, methylated CpGs; representative of three individual experiments with 5-azacytidine (AZA)-treated or untreated from one individual with SAT hypertrophy

To further explore the mechanisms determining *ZNF423* expression in SVF pre-adipocytes, we performed bisulphite sequencing of the entire enhancer CpGi in three individuals who had the smallest sized subcutaneous adipocytes, and an equal number of individuals exhibiting the largest adipocyte size. All individuals were non-obese and had a similar BMI. This revealed massively increased methylation levels at two subregions of the CpG enhancer island in the latter individuals (Fig. 7a). The R2 subregion showed >90% methylation at CpG dinucleotides 30 and 33 in participants with subcutaneous adipocyte hypertrophy, compared with <20% in those with smaller adipocytes (Fig. 7b). The R3 subregion revealed that methylation at CpG nucleotides 54, 55, 56, 57, 60 and 62 is >90% and <45% respectively, in individuals with and without adipose cell hypertrophy. In addition, 5-azacytidine treatment of pre-adipocytes from individuals with adipose cell hypertrophy led to a greater than twofold increase in expression of *ZNF423* (Fig. 7c) and its downstream target gene *PPARγ* (Fig. 7d). 5-Azacytidine strongly decreased the methylation level at CpG positions 54, 55, 56, 57, 60 and 62 of the R3 subregion, consistent with the important role of these CpGs in regulating *ZNF423* expression (Fig. 7e). Methylation at regions R1 and R2 was not affected by 5-azacytidine (data not shown).

Taken together, these data indicate a non-permissive transcriptional state at the *ZNF423* locus in SVF precursor cells from individuals who develop inappropriate subcutaneous adipose cell hypertrophy, a marker of impaired adipogenesis.

## Discussion

Previous studies have demonstrated the importance of the *Pparγ* transcriptional activator ZFP423 in regulating pre-adipocyte determination [17], and shown that *Zfp423* expression identifies committed pre-adipocytes [19]. Thus, *Zfp423* is crucial for the initial formation of white adipocytes and, importantly, also plays a later role in maintaining the energy-storing phenotype of white adipose cells [18]. Epigenetic mechanisms have been linked to the transcriptional regulation of *Zfp423* exerted by ZFP521. ZFP521 binds the promoter and intronic regions of *Zfp423* and represses its expression by promoting histone modifications. These findings support growing evidence that lineage determination of multipotent MSCs to the adipocyte lineage is also epigenetically regulated [30]. Consistent with this, we show here that *Zfp423* was transcribed in 3T3-L1 pre-adipose cells but not in NIH-3T3 non-pre-adipose fibroblasts. Furthermore, we identified a large CpGi at the *Zfp423* promoter and report, for the first time, that *Zfp423* expression in 3T3-L1 cells is accompanied by an extensive demethylation of this *Zfp423* region, followed by decreased nucleosome occupancy.

This implicated a causal relationship between the different epigenetic profile and *Zfp423* transcription in 3T3-L1 and NIH-3T3 cells. Consistent with this, we found no DNA sequence variation at the *Zfp423* promoter in the two cell types. Luciferase assays provided formal proof that methylation directly represses *Zfp423* promoter function in vitro. In addition, exposure to the demethylating agent 5-azacytidine simultaneously caused *Zfp423* promoter demethylation and rescued *Zfp423* transcription in intact NIH-3T3 fibroblasts. Thus, promoter methylation is an important regulator of the differential transcription of *Zfp423* in non-pre-adipose and pre-adipose fibroblasts.

DNA methylation inhibits gene expression by at least two mechanisms. First, cytosine methylation may directly inhibit the association of DNA-binding factors [31, 32]. Second, proteins that recognise methylated CpG sites may recruit transcriptional corepressor molecules, including histone modification and chromatin remodelling enzymes, and cause a transcriptionally repressed chromatin state [31, 32]. Here, MNase digestion assays revealed that, in the pre-adipose 3T3-L1 fibroblasts, CpG demethylation of the *Zfp423* promoter was accompanied by nucleosome repositioning in an open chromatin state, which may contribute to *Zfp423* active transcription. The ability of 5-azacytidine to induce this nucleosome repositioning suggests that, in NIH-3T3 cells, demethylation of the *Zfp423* promoter may trigger chromatin remodelling in a transcriptionally active conformation, thereby inducing *Zfp423* expression. Therefore, although we did not investigate a direct association of transcriptionally relevant DNA-binding factors to methylated cytosines, CpG methylation-triggered chromatin condensation appears to be an important mechanism for maintaining methylated *Zfp423* promoter silencing.

Previous studies have demonstrated that *Zfp423* transcription is essential for pre-adipocyte commitment, enabling further adipogenic differentiation [17–19]. In line with this, we showed that the effect of 5-azacytidine on *Zfp423* promoter epigenetics and active gene transcription was followed by rescue of the differentiation capacity of the NIH-3T3 fibroblasts, as revealed by a robust rise in *Pparγ*, *Fabp4*, *Adipoq* and *Glut4* levels. Oil Red O accumulation in NIH-3T3 cytoplasm was also increased following exposure to 5-azacytidine. Therefore, in the model we now propose, commitment of an adipocyte precursor cell is accompanied by acquisition of a specific chromatin epigenetic signature of the *Zfp423* locus [17, 33]. Importantly, as shown here, these events appear to be reversible. Indeed, exposure of the uncommitted NIH-3T3 cell to an epigenetic agent, i.e. 5-azacytidine, partly reprogrammed the epigenetic signature at the *Zfp423* promoter, favouring commitment and adipogenesis. However, 5-azacytidine does not make the NIH-3T3 cells an in vitro model of spontaneous adipogenesis (data not shown). This is not surprising because *Zfp423*, identified as a major determinant of pre-adipocyte commitment, is not responsible for the early phase of adipogenesis. At this stage, only ectopic expression of CCAAT/enhancer-binding protein β provides a surrogate for the requirement for 3-isobutyl-1-methylxanthine in the adipogenic differentiation of NIH-3T3 cells [34]. It is possible that further work will generate novel opportunities to overcome the restricted subcutaneous adipogenesis that is predictive of type 2 diabetes.

Studies by Bowers and co-workers [35] have demonstrated stem cell commitment to the adipocyte lineage by 5-azacytidine inhibition of DNA methylation. They also provided evidence supporting the role of BMP4 signalling in adipocyte lineage determination induced by 5-azacytidine [36]. Additional studies have revealed that increased expression and secretion of BMP4, a key molecule in the adipogenic microenvironment as it is also secreted by mature adipose cells [37], correlate with the capacity of MSCs to undergo adipogenic differentiation [36, 38]. Importantly, BMP4 has been shown to enable nuclear entry of ZFP423 by dissociating the cytoplasmic WISP2–ZFP423 protein complex, which retains ZFP423 in the cytosol [20], thereby activating *Pparγ* transcription. Silencing of *Zfp423* completely prevents the induction of *Pparγ* and other adipogenic marker genes in BMP4-treated cells, showing that ZFP423 is crucial for *Pparγ* activation and for the ability of BMP4 to induce *Pparγ* transcription [20].

Here, we demonstrate, for the first time, that BMP4 also causes demethylation of the *Zfp423* promoter, which is sufficient to commit otherwise non-adipogenic cells to the adipogenic lineage. Thus, convergence of BMP4 signalling on *Zfp423* enables its action on pre-adipocyte determination through multiple mechanisms, including epigenetic modifications at key genes and nuclear import of ZFP423.

Interestingly BMP2, a BMP4 homologue, only slightly reduces *Zfp423* expression in NIH-3T3 cells, probably because, as previously reported [39, 40], the BMP2 target and *Zfp423* inhibitor ZFP521 is expressed in these cells. Addison et al have reported that BMP2-induced commitment of MSCs to the adipose lineage is likely to be suppressed by ZFP521 through direct inhibition of *Zfp423*, providing a potential explanation for why BMP2 responses are predominantly osteogenic [30].

Detailed analysis of BMP4 action on *Zfp423* transcription revealed that BMP4-induced demethylation selectively involved the CpG dinucleotide at position −1016 from the *Zfp423* transcription start site. Interestingly, methylation at this site was invariably inhibited after 5-azacytidine treatment of NIH-3T3 cells. These novel findings suggest a functional relevance of the −1016 dinucleotide. Subsequent mutagenesis experiments demonstrated that, while not affecting the in vitro function of the demethylated *Zfp423* promoter, the introduction of a point mutation at the −1016 CpG position prevented the methylation-dependent silencing of *Zfp423* transcription. Accordingly, we suggest that the regulatory effect of methylation at the *Zfp423* promoter is dependent not only on the quantitative dimension of the methylation events, but also on the specific promoter region that is affected.

Restricted adipogenesis in human SAT is determined by impaired adipocyte precursor cell commitment [12, 13, 16] and results in hypertrophy of adipocytes in the SAT [17, 41]. Our recent studies have shown that markers of restricted subcutaneous adipogenesis with inappropriate adipose cell hypertrophy are associated with a family history of type 2 diabetes [16, 42] and are also present in non-obese individuals with type 2 diabetes [43]. The relevance of our mechanistic findings in the NIH-3T3/3T3-L1 cell model to human adipose tissue dysfunction is underlined by our results in human adipocyte precursors revealing that expression of the *Zfp423* human orthologue *ZNF423* [29] negatively correlates with the cell size of mature adipocytes. Hence, in the same individuals, low *ZNF423* expression in SVF pre-adipocytes is a marker of impaired adipogenesis leading to inappropriate hypertrophy of mature subcutaneous adipocytes and causally contributing to it by interfering with the adipogenic commitment and differentiation of precursor cells.

Taken together, our present results suggest that the restricted subcutaneous adipogenesis associated with insulin resistance [6] and a family history of type 2 diabetes [16, 42, 43] may be due to dysfunctional epigenetic regulation rather than conventional DNA risk genes.

Secretion of BMP4 by mature adipose cells is positively correlated with adipose cell size, and we have suggested that this is part of positive feedback in the tissue to enhance the commitment and differentiation of new precursor cells to prevent inappropriate hypertrophy [37, 44]. Here, we provide a molecular basis for the effect of BMP4 in enhancing adipogenesis, although secretion of BMP4 antagonists, in particular Gremlin 1 in humans [37], is increased in hypertrophic obesity and prevents the expected positive effect of BMP4 on adipogenesis.

The overall structure of the regulatory regions of human *ZNF423* and mouse *Zfp423* is quite different [29]. However, we observed massive hypermethylation at distinct CpG dinucleotides in the central island serving as a promoter enhancer in human *ZNF423*. Hypermethylation of this island has previously been shown to silence the gene in human leukaemia cells [29]. The position of the regulatory CpG dinucleotides, which are targeted by methylation events in leukaemia and adipocyte precursor cells, differ, probably reflecting tissue specificity [45]. However, as demonstrated in the leukaemia cells, methylation at the pre-adipocyte ZNF423 central enhancer island may also feature a repressive function as its presence closely correlated with reduced *ZNF423* expression in the adipocyte precursor cells. We propose, therefore, that changes in the methylation profile at the regulatory region account for the reduced *ZNF423* expression observed in hypertrophic adipose tissue. Indeed, 5-azacytidine treatment of pre-adipocytes isolated from individuals with adipose cell hypertrophy rescues both the hypomethylated and the permissive state at specific CpG enhancer region dinucleotides, as well as ZNF423 expression.

Thus, based on our findings, methylation at the *ZNF423* regulatory region and its expression can be targeted both pharmacologically (e.g. 5-azacytidine) and by changes in the adipose tissue microenvironment (e.g. changes in BMP4 abundance or signalling). Expansion of this work may generate attractive and novel opportunities to overcome the restricted subcutaneous adipogenesis and prevent inappropriate adipose tissue hypertrophy and its negative consequences on metabolism and risk of type 2 diabetes.

## Electronic supplementary material

ESM (PDF 320 kb)

## Abbreviations

BMP4: Bone morphogenetic protein 4
MNase: Micrococcal nuclease
MSC: Mesenchymal stem cell
NUC1/2: Nucleosome 1/2
qPCR: Quantitative real-time PCR
SAT: Subcutaneous adipose tissue
SVF: Stromal vascular fraction
WISP2: WNT-inducible secreted protein 2
ZFP: Zinc finger protein
