# Supplementary material for: Epigenetic modifications of the Zfp/ZNF423 gene control murine adipogenic commitment and are dysregulated in human hypertrophic obesity
Source: Diabetologia. 2017 Oct 24;61(2):369–80. doi: 10.1007/s00125-017-4471-4 (PMC6448963; doi:10.1007/s00125-017-4471-4)
Supplement: Supplementary file 1 — (PDF 320 kb) [file 125_2017_4471_MOESM1_ESM.pdf]

# Electronic supplementary material (ESM)

## ESM Methods

**Cell culture and adipocyte differentiation.** For adipocyte differentiation, cells were grown to confluence in medium containing 10% FCS. Two days after reaching confluence, the cells were cultured in DMEM supplemented with a differentiation cocktail containing 5μg/ml insulin, 0.5mmol/l 3-isobutyl-1-methylxanthine, 1mol/l dexamethasone, 1 μmol/l rosiglitazone and 10% FBS for 2 days. Forty-eight hours after induction, cells were maintained in DMEM containing 5μg/ml insulin and 10% FBS until they were ready for collection. The NIH-3T3 cells were cultured in the presence or the absence 5-Azacytidine (5μmol/l) for 6h before the administration of differentiation cocktail. Lipid accumulation of mature adipocytes was determined by Oil Red O staining as reported in [13]. The cells were incubated for 60 min at RT in Oil Red O staining solution. Images were taken using an Olympus microscope system (Center Valley, PA, USA). For quantification, absorbance was measured at 510 nm using a spectrophotometer (Beckman, CA, USA).

## ESM Tables

| Gene | Forward/Reverse | Primer (5’ to 3’) |
|---|---|---|
| *Pparγ2* | Forward | CAGTGGAGACCGCCCAGGCT |
| *mRNA* | Reverse | TGGAGCAGGGGGTGAAGGCT |
| *Fabp4* | Forward | TCTCACCTGGAAGACAGCTCC |
| *mRNA* | Reverse | GCTGATGATCATGTTGGGCTTGG |
| *Glut4* | Forward | CAGAAGGTGATTGAACAGAG |
| *mRNA* | Reverse | AATGATGCCAATGAGAAA |
| *Adipoq* | Forward | GTGACGACACCAAAAGGGCTC |
| *mRNA* | Reverse | TCCAACCTGCACAAGTTCCC |
| *Ppia* | Forward | GCAGACAAAGTTCCAAAGACAG |
| *mRNA* | Reverse | CACCCTGGCACATGAATCC |
| *Zfp423* | Forward | GGTTTTATTATGTGTTTTTGTAGTGTA |
| *mRNA* | Reverse | ATATCCCTCAACTCAACCTACTTAA |
| *ZNF423* | Forward | AGGCCTAGAAGGAGAGCCAG |
| *mRNA* | Reverse | TCGTCATCACCATCTCCAGG |
| *Zfp423 NUC 1* | Forward | CCCGCACGGGCCTGTTA |
| *MNase* | Reverse | CTCTGACAGCACTGGGCA |
| *Zfp423 NUC 2* | Forward | TGTGGCCGGACGCCTG |
| *MNase* | Reverse | CCTTCTCCTCCGCCCCTTG |
| *Zfp423 CTRL R* | Forward | GCCCGAGGGCAGGCA |
| *MNase* | Reverse | GCACGGGCATTGCTCAG |
| *Zfp423* | Forward | GGTTTTATTATGTGTTTTTGTAGTGTA |
| *Bisulfite* | Reverse | ATATCCCTCAACTCAACCTACTTAA |
| *ZNF423* | Forward | GAGAGGAGGAAGAAATTTAGGGTGGGGTG |
| *Bisulfite* | Reverse | ACTCAAAACAATCCTCAATACCTAAAAAATAC |

**ESM Table 1.** List of primers used in this study

## ESM Figures

ESM Fig.1

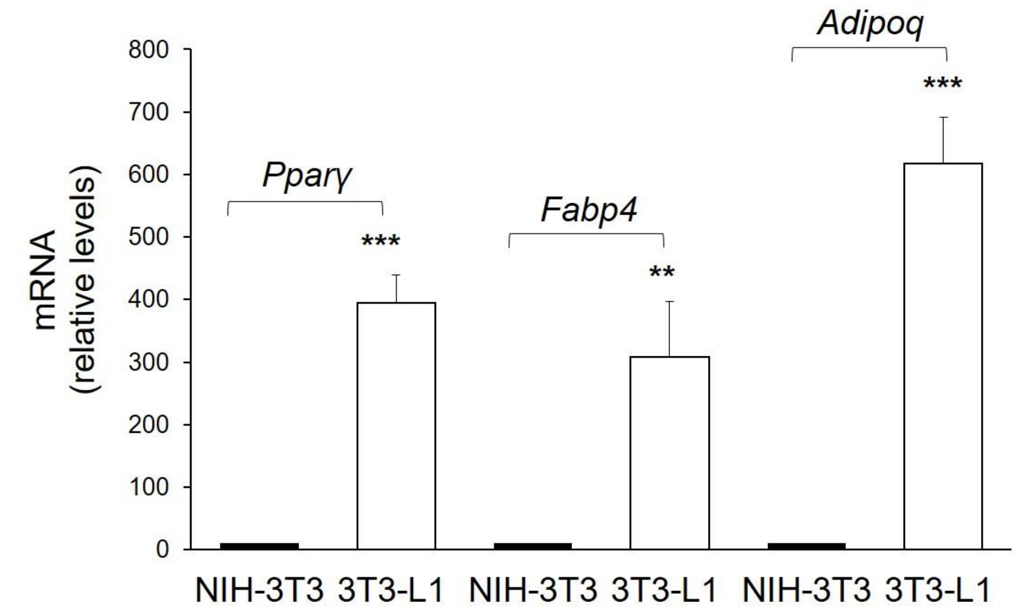


**ESM Fig.1. Adipocyte marker genes in NIH-3T3 and 3T3-L1 cells.** Total RNA was isolated from the cells and the relative mRNA levels of *Pparγ, Fabp4,* and *Adipoq* were determined by qPCR. Data normalisation has been performed using the housekeeping *Ppia* gene as internal control. Results are the means ± SD of three independent experiments. Statistical significance was established by 2-tail Student's t-test (**$p$<0.01, and ***$p$<0.001).

ESM Fig.2

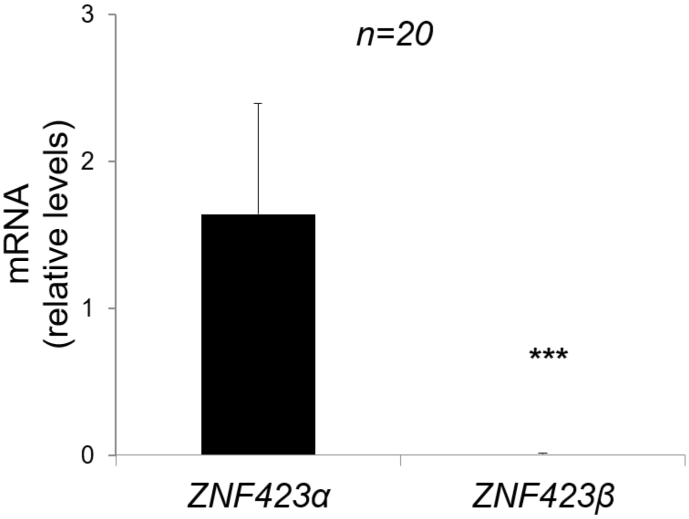


**ESM Fig.2. Expression of *ZNF423α* and *β* isoforms in SVF preadipocytes.** Expression of *ZNF423α* (NM_015069) and *ZNF423β* (NM_001271620) mRNA was measured by qPCR. Data normalisation has been performed using the housekeeping *Ppia* gene (2-ΔCT*1000) as internal control. Results are the means ± SD, with *n* equal to the number of sample analysed. Statistical significance was established by 2-tail Student's t-test (***$p$<0.001).
